# Machine learning‐based phenotypic screening for postmitotic growth inducers uncover vitamin D3 metabolites as small molecule ribosome agonists

**DOI:** 10.1111/cpr.13214

**Published:** 2022-04-12

**Authors:** Zongmin Jiang, Liping Zhang, Ziyue Yao, Wenhua Cao, Shilin Ma, Yu Chen, Lu Guang, Zipeng Zheng, Chunwei Li, Kang Yu, Ng Shyh‐Chang

**Affiliations:** ^1^ State Key Laboratory of Stem Cell and Reproductive Biology, Institute for Stem Cell and Regeneration, Institute of Zoology Chinese Academy of Sciences Beijing China; ^2^ Beijing Institute for Stem Cell and Regenerative Medicine Beijing China; ^3^ University of Chinese Academy of Sciences Beijing China; ^4^ Department of Clinical Nutrition, Peking Union Medical College Hospital, Chinese Academy of Medical Sciences (CAMS) Peking Union Medical College (PUMC) Beijing China

## Abstract

**Objectives:**

To restore tissue growth without increasing the risk for cancer during aging, there is a need to identify small molecule drugs that can increase cell growth without increasing cell proliferation. While there have been numerous high‐throughput drug screens for cell proliferation, there have been few screens for post‐mitotic anabolic growth.

**Materials and Methods:**

A machine learning (ML)‐based phenotypic screening strategy was used to discover metabolites that boost muscle growth. Western blot, qRT‐PCR and immunofluorescence staining were used to evaluate myotube hypertrophy/maturation or protein synthesis. Mass spectrometry (MS)‐based thermal proteome profiling‐temperature range (TPP‐TR) technology was used to identify the protein targets that bind the metabolites. Ribo‐MEGA size exclusion chromatography (SEC) analysis was used to verify whether the ribosome proteins bound to calcitriol.

**Results:**

We discovered both the inactive cholecalciferol and the bioactive calcitriol are amongst the top hits that boost post‐mitotic growth. A large number of ribosomal proteins' melting curves were affected by calcitriol treatment, suggesting that calcitriol binds to the ribosome complex directly. Purified ribosomes directly bound to pure calcitriol. Moreover, we found that calcitriol could increase myosin heavy chain (MHC) protein translation and overall nascent protein synthesis in a cycloheximide‐sensitive manner, indicating that calcitriol can directly bind and enhance ribosomal activity to boost muscle growth.

**Conclusion:**

Through the combined strategy of ML‐based phenotypic screening and MS‐based omics, we have fortuitously discovered a new class of metabolite small molecules that can directly activate ribosomes to promote post‐mitotic growth.

## Graphical abstract

High‐throughput drug screening based on machine learning‐driven recognition of human myoblast‐derived myotubes and their three‐dimensional volumes, to assay for postmitotic growth.

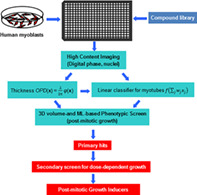

## 1 INTRODUCTION

Tissue ageing may cause tissue damage and degeneration, in some cases leading to ageing‐related diseases that involve tissue/cell atrophy, such as sarcopenia, cachexia, osteoporosis, pancreatic atrophy and Alzheimer's disease.^1^, ^2^, ^3^, ^4^, ^5^ In order to restore tissue growth without increasing the risk for cancer, there is a need to identify small molecule drugs that can increase cell growth, without increasing cell proliferation. While there have been numerous high‐throughput drug screens for cell proliferation, there have been few screens for postmitotic anabolic growth, for example, in skeletal muscles. Skeletal muscle is composed of postmitotic myofibres.^6^ Skeletal muscle mass and strength decrease with advancing age in sarcopenia, as net muscle protein synthesis becomes dramatically decreased.^4^, ^7^, ^8^, ^9^ In two‐dimensional (2D) culture in vitro, human skeletal muscle myoblasts are able to exit the cell cycle, fuse and differentiate into postmitotic multinucleated myotubes, where the length and diameter distributions of myotubes could be indicators of postmitotic growth. However, aside from a few screens for drugs that promote muscle contraction in response to chemical stimulation^10^ and the rescue of dystrophin deficiency,^11^ there have been few if any drug screens that exclusively focus on postmitotic growth or cell volume. Due to the stochasticity of myoblast fusion and myotube directionality, automated and accurate extraction of relevant morphological parameters for high‐throughput drug screening had remained elusive.

In the field of drug screening, the significant drop in newly approved drugs in recent decades has been partially attributed to failures in the target‐based screening model. Evaluation of new drugs approved by FDA has revealed that the number of successful drugs from phenotypic screens exceeded those discovered through molecular target‐based screens, despite the overwhelmingly larger amounts of investments into target‐based projects over the same period.^11^ This surprising result has contributed to growing interest in phenotypic screens for drug development. Nevertheless, phenotypic screening has its own problems. Lacking a well‐defined target(s) makes it difficult to further optimize the drugs, identify molecular biomarkers and select patient subpopulations for clinical trials. An emerging technology that combines the strengths of phenotypic screens and target‐based screens is mass spectrometry (MS)‐based thermal proteome profiling (TPP). TPP enables an unbiased and comprehensive search of the targets of a small molecule drug across the entire proteome, by assuming that drug‐binding alters its target protein's structure and thus thermal stability in solution, which can be revealed by comparing proteins' abundance in solution, before and shortly after drug treatment, over a temperature range.^12^, ^13^ Another advantage of TPP is that it can discover targets in living cells and tissues without requiring compound labelling for pulldowns.^13^, ^14^ Thus, TPP affords a high‐throughput strategy to fuse the advantages of phenotypic screens with target‐based drug discovery.

Here our platform of ML‐based phenotypic screens for postmitotic growth combined with MS‐based TPP proteomic target discovery, revealed that vitamin D3 metabolites promote postmitotic muscle growth by directly binding and activating ribosomes. The inactive vitamin D3 (cholecalciferol) needs to be metabolized into the inactive 25‐hydroxyvitamin D3 (25(OH)D or calcifediol) in the liver, then transported to the kidney where it is finally metabolized to the bioactive form 1,25‐dihydroxyvitamin D3 (1,25(OH)2D or calcitriol).^15^ Vitamin D3 deficiency is common among older adults and has been linked to muscle weakness and subsequent sarcopenia, potentially leading to increased risk of chronic pain, frailty, falls and death.^16^, ^17^, ^18^ Calcitriol exerts genomic effects through the vitamin D nuclear hormone receptor (VDR) to control gene transcription, but it is also widely‐speculated to possess rapid and nongenomic effects, although this noncanonical mechanism(s) had remained unclear.^19^, ^20^, ^21^ Moreover, VDR expression in muscle tissue is progressively reduced during development and ageing,^22^, ^23^ with very low levels remaining in adult skeletal muscle.^24^ Our discovery that postmitotic growth can be enhanced by both the “inactive” cholecalciferol and “active” calcitriol, each with VDR *K*_d_'s that are orders of magnitude apart, corroborates the importance of noncanonical, VDR‐independent mechanisms.

In summary, our ML‐based phenotypic screen of 3D cell volumes revealed that vitamin D3 metabolites are top hits in boosting postmitotic growth. MS‐based TPP technology further revealed that the ribosomal complex was a novel target of calcitriol. Ribo Mega size exclusion chromatography (SEC) orthogonally confirmed that calcitriol could directly bind to purified ribosome complexes. Using functional assays, we found that one of calcitriol's nongenomic effects is to act as a small molecule ribosome agonist that directly promotes protein synthesis and elevates myofibrillar protein expression, suggesting a novel mechanism for why vitamin D3 metabolites are one of the most effective small molecule metabolites to improve muscular mass and strength. Our platform's findings suggest that calcitriol and related vitamin D3 metabolites may be archetypal members of a new class of ribosome agonists, with implications for inducing postmitotic growth and treating degenerative diseases of ageing without promoting cancer.

## 2 METHODS

### 2.1 Cell culture

Commercial primary adult human myoblasts (Gibco) were expanded in Dulbecco's modified Eagle medium (DMEM) growth medium supplemented with 20%FBS (BIOIND) and 1% penicillin–streptomycin (Gibco) at 37°C and 5%CO_2_. Human myoblasts (100% confluency) were maintained in a serum‐free medium (1% KSR [KnockOut Serum Replacement, Gibco] in DMEM, 1% l‐glutamine [Gibco] and 1% penicillin–streptomycin) for 7 days to induced myotube formation.

### 2.2 High‐content, high‐throughput screening

At Day 0, 700 human myoblasts/well were seeded in a gelatin‐coated 384‐well μClear microplate (Greiner) and maintained in specialized growth medium. Twenty‐four hours later (Day 1), until 90% cell confluence, growth medium was replaced with 1%KSR medium after 1 wash with PBS (no Ca^2+^ and Mg^2+^, pH 7.4), and then treated with 1 μM of each compound (Target Molecule Corp.) for 7–9 days at 37°C and 5%CO_2_. dimethyl sulfoxide (DMSO) 0.1% vol/vol was used as the vehicle control.

Images were acquired with a PerkinElmer Operetta CLS High Content Screening System with a ×10 high NA objective. Nuclear and whole cell images were analysed for Hoechst 33342 staining and Digital Phase Contrast signals to determine nuclei numbers and 3D cell volumes respectively, at Days 3, 5, 7 and 9. Images were analysed by using Harmony 4.6 software, and programmed for automated segmentation of nuclei and myotubes. First, nuclei were defined and selected by an optimal area and roundness threshold. Whole myotubes were defined using cell area, length and intensity thresholds, after recognition with linear classifier‐based machine learning (ML) algorithms trained and optimized in house.

Specifically, multinucleated myotubes were extracted and calculated by optimizing for specific parameters, including the min/max myotube area, min/max myotube length and minimum intensity thresholds. Finally, the multinucleated myotube area (%) was used as a parameter to identify positive drug hits.

### 2.3 Fusion index

Fusion index was assessed by calculating the ratio of multinucleated (>2) myotube nuclei to the total number of nuclei. In brief, human myoblasts were cultured with 1% KSR medium supplemented with 0.01% DMSO vehicle or 100 nM calcitriol (Selleckchem Inc.), these myoblasts were differentiated into and formed multinucleated myotubes. On Day 7 after differentiating, myotubes were washed with PBS and fixed with 2%PFA. Myotubes were incubated with anti‐MHC/MF20 (DSHB, 2.5 μg/ml), followed by secondary antibody anti‐mouse IgG Alexa Fluor 488 (1:500; Thermo Fisher Scientific). DAPI (1:1000; Thermo Fisher Scientific) staining was used for nuclei counting. Stained cells were imaged with a Nikon fluorescence microscope, and at least 20 images per group were included in the statistics.

### 2.4 Quantitative real‐time polymerase chain reaction

Quantitative real‐time polymerase chain reaction (RT‐PCR) was performed using a LightCycler480 II sequence detection system (Roche Applied Science). RNA was extracted by TRIzol (Takara) and reverse transcribed with PrimeScript 286 RT (Takara), and quantitative PCR was conducted by using KAPA SYBR FAST qPCR Kits (KAPA Biosystem) following the manufacturer's instructions. All primer sequences were as reported previously.^25^

### 2.5 Western blots

Protein extracts were prepared with RIPA lysis buffer (89901; Thermo Fisher Scientific) with protease inhibitor cocktail (Thermo Fisher Scientific). All lysates were quantified by BCA protein assay (Thermo Fisher Scientific). Ten microgrammes of protein from each sample were electrophoresed on 8% sodium dodecyl sulphate–polyacrylamide gel electrophoresis and transferred to nitrocellulose membranes (Merck Millipore). Blots were incubated with primary antibodies: MHC/MF20 (AB2147781; DSHB, 0.5 μg/ml), MyoD1 (sc‐377 460; 1:1000; Santa Cruz), myogenin/MYOG (sc‐52903; 1:1000; Santa Cruz) and GAPDH (2118; 1:2000; Cell Signalling), then probed with the secondary antibody anti‐mouse IgG HRP or anti‐rabbit IgG HRP (Thermo Fisher Scientific). HRP‐based detection was performed using an iBright FL1000 Imaging System (Invitrogen).

### 2.6 Thermal proteome profiling

Protein samples were prepared from control (0.01% DMSO) and calcitriol‐exposed myotubes. For each group, 2 × 10^7^ myoblasts were seeded in two 15 cm cultured dishes. After 24 h of culturing, growth medium was replaced with 1% KSR medium after one wash with PBS, to induce terminal differentiation. After 72 h of differentiation, cells were incubated with fresh 1% KSR medium supplemented with 100 nM calcitriol or 0.1% (vol/vol) DMSO vehicle for 3 h. Cells were washed with PBS, digested with trypsin, collected, and centrifuged at 300*g* for 5 min at 4°C. Cell pellets were resuspended with 1.2 ml of ice‐cold PBS containing EDTA‐free protease inhibitors, then aliquoted and centrifuged at 300*g* for 3 min at 4°C to pellet the cells. Cell samples were heated in parallel for 3 min at 37°C, 41°C, 44°C, 47°C, 50°C, 53°C, 56°C, 59°C in the Veriti 96‐well Thermal Cycler (Applied Biosystems), followed by 3‐min incubation at room temperature. Immediately after incubation, all cell samples were put on ice. Cell samples were lysed via 5 cycles of snap freezing in liquid nitrogen for 30 s and thawing in a 25°C water bath for 30 s, followed by mechanical vortex. Protein solutions were isolated after centrifugation and pelleting of denatured or insoluble proteins (10,000*g*, 20 min, 4°C), and the protein concentration was determined by BCA assays.

### 2.7 Digestion, TMT labelling, and fractionation

For peptide extraction, 120 μg of TPP protein samples (37°C, 44°C, 47°C, 50°C, 53°C, 56°C, 59°C) were incubated with 100 mM DTT and 200μl of 8 M urea for 1 h at 56°C, followed by 50 mM IAA buffer and 100 mM TEAB buffer incubation for 30 min at room temperature. Furthermore, proteins were digested with 2.5 μg trypsin (Promega) in 45 μl 50 mM TEAB buffer for 18 h at 37°C. Peptides were labelled by TMTpro 16plex Lable (Thermo Fisher Scientific) following the manufacturer's instructions, desalted using Sep‐Pak C18 column (Waters) and dried in vacuo. Peptides were redissolved in 10 mM ammonium formate pH 10.0, then separated into fifth fractions by high pH gradient separation (from 0% to 90% of 10 mM ammonium formate in 90% ACN for 66 min and held for 5 min) using 1290UPLC system (Agilent Corp.) connected to a BEH C18 column (2.1 × 150 mm, 1.7 μm, 300 Å; Waters). The flow rate was 250 μl/min, and column temperature was kept at 50°C. Each fraction was collected and dried in vacuo.

### 2.8 LC–MS/MS

For liquid chromatography–mass spectrometry (LC–MS) proteomic data collection, the fractions resolved in 0.1% formic acid were separated by nanoLC, and analysed using EASY‐nLCTM 1200 system‐coupled to a Q‐Exactive‐HF‐X (Thermo Fisher Scientific) mass spectrometer, according to previously reported methods.^13^ In brief, 4 μl peptide sample was loaded with a flow rate of 300 nL/minute in a Phenomenex analytical column (75 μm × 20 cm, Aqua 3 μm C18 125 Å), followed by gradient separation from 1% to 37% 0.1% formic acid in CAN for 63 min. At 65‐min separation, the gradient was increased to 95% 10 mM ammonium formate in 90% CAN and held for 5 min.

The Q‐Exactive‐HF‐X mass spectrometer switched automatically between MS and MS/MS acquisition. For each experimental cycle, full‐scan MS spectra (*m*/*z* 350–1800) was acquired at a mass resolution of 60 K, setting an automatic gain control (AGC) target of 3 × 10^6^, and a maximum injection time of 20 ms, to acquire 10 sequential energy collisional dissociation MS/MS scans with a resolution of 45.0 K in Orbitrap. Subsequently, to set other parameters: an intensity threshold of 1 × 10^4^, a maximum filled time of 100 ms, AGC target of 2 × 10^5^, the isolation window of 0.7 *m*/*z*. Ions with charge states 2+, 3+ and 4+ were fragmented with the normalized collision energy of 35%. Tandem mass spectra were extracted by Proteome Discoverer 2.4 software (Thermo Fisher Scientific), and protein identification was carried out using SEQUEST traced to search the UniProtKB (*Homo sapiens*) database. Search tolerances were specified to 10.0 PPM for the precursor ion and 0.02 Da for a fragment mass. Carbamidomethyl of cysteine and TMT pro plex of lysine and the N‐terminus were specified in SEQUEST as fixed modifications. Methionine oxidation was set as a variable modification.

### 2.9 Protein synthesis assays

Human myoblasts in six‐well plates (100% confluency) were incubated in 1% KSR medium. After 24‐h differentiation, the cells were incubated in fresh 1% KSR medium containing 50 μg/ml CHX (cycloheximide, to block further protein synthesis), in the presence or absence of 100 nM calcitriol. Importantly, CHX was added to the cells 1 h before calcitriol. After CHX ± calcitriol incubation, the human myoblasts were washed with PBS and replaced with 1% KSR ± calcitriol for further differentiation. After terminal differentiation, the cells were washed with PBS and collected for Western blotting. Western blots for myosin heavy chain (MHC), MYOD1 and MYOG, were used to measure steady state protein levels, as described above.

Nascent protein synthesis was quantified by measuring incorporated *O*‐propargyl‐puromycin (OP‐puro) into polypeptides. Briefly, 4000 human myoblasts (each well) were seeded in a gelatin‐coated 96‐well μClear microplate (Greiner), then incubated in 1% KSR medium for myotube differentiation. After 72‐h differentiation, the cells were incubated with 10 μM OP‐puro (MedChem) and 0.01% DMSO (as the negative control) or 100 nM calcitriol for 0.5 h at 37°C and 5%CO_2_, while 1% KSR without OP‐puro served as the unlabeled control. Immediately after incubation, a click reaction was performed between click‐iT Plus OPP Alexa Fluro 594 and the OP‐Puro, according to the manufacturer's protocol (C10457; Invitrogen). The stained cells were imaged by Nikon fluorescence microscopy. Average fluorescence intensity was qualified in ImageJ, the data were obtained from at least 10 images from three independent experiments.

Western blotting for puromycin was also used to quantify nascent protein synthesis. Human myoblasts (2 × 10^6^) in a six‐well plate were grown, differentiated and incubated with 10 μM OP‐puro ±0.01% DMSO or 100 nM calcitriol as described above. Immediately after incubation, the cells were washed with cold PBS three times, followed by protein lysis and BCA quantification. Blots were incubated with anti‐puromycin clone 12D10 (1:3000; Sigma).

### 2.10 Ribo Mega‐SEC and uHPLC

Human myoblasts in two 15‐cm dishes were differentiated into mature myotubes for 96 h, then incubated with 1% KSR medium containing 50 μg/ml CHX for 5 min at 37°C and 5% CO_2_. After incubation, the cells were washed with cold PBS and 1 ml polysome extraction buffer, incubated for 15 min on ice, scraped and collected by centrifuging at 17,000*g* for 10 min at 4°C. Cell lysates were removed and filtered through a 0.45 μm Ultrafree‐MC HV filter column (Millipore), followed by centrifuging at 12,000*g* for 2 min at 4°C. Lysates were quantified by BCA protein assays. The polysome extraction buffer comprises of 20 mM Hepes‐NaOH (pH 7.4) 130 mM NaCl, 10 mM MgCl_2_, 1% CHAPS, 0.2 mg/ml heparin, 2.5 mM DTT, 50 μg/ml CHX, EDTA‐free protein and 20 U RNAase inhibitor.

Ribo Mega‐SEC uHPLC is required for efficient separation and collection of polysomes, 80S ribosomes, 60S and 40S ribosomal subunits.^26^ One Agilent Bio SEC‐52000 Å column (7.8 × 300 mm with 5‐μm particles) was installed in a Dionex Ultimate 3000 Bio‐RS uHPLC system (Thermo Fisher Scientific), and equilibrated with two column volumes of filtered SEC buffer. SEC buffer consists of 20 mM Hepes‐NaOH (pH 7.4) 0.5 M NaCl, 10 mM MgCl_2_, 0.3% CHAPS, 0.2 mg/ml heparin, 2.5 mM DTT, 50 μg/ml CHX, EDTA‐free protein and 20 U RNAase inhibitor. After 30‐min baseline equilibrium, 100 μl lysates (~200 μg) was injected and separated with SEC buffer, in the absence or presence of 20 μM calcitriol. The flow rate was 0.8 ml/min (runs of ~15 min), while the temperature of columns and protein samples were maintained at 6°C. The chromatogram was monitored by measuring UV absorbance at 214, 260, and 280 nm with 2.5 Hz of data collection rate by the Diode Array Detector. The chromatograms (protein ± calcitriol) were merged and processed by using Chromeleon 7.0 software.

### 2.11 Statistical analysis

All experiments were performed ≥3 times in triplicate and data are represented as mean value ± *SEM* for statistical comparisons. Statistical analysis was performed by using GraphPad Prism 9.0 and Microsoft Excel. Significance of differences was assessed by a paired two‐tailed Student's *t‐*test to compare two groups. A value of *p* < 0.05 was considered statistically significant.

## 3 RESULTS

### 3.1 Identification of natural compounds that promote muscle volumetric growth

**FIGURE 1:**
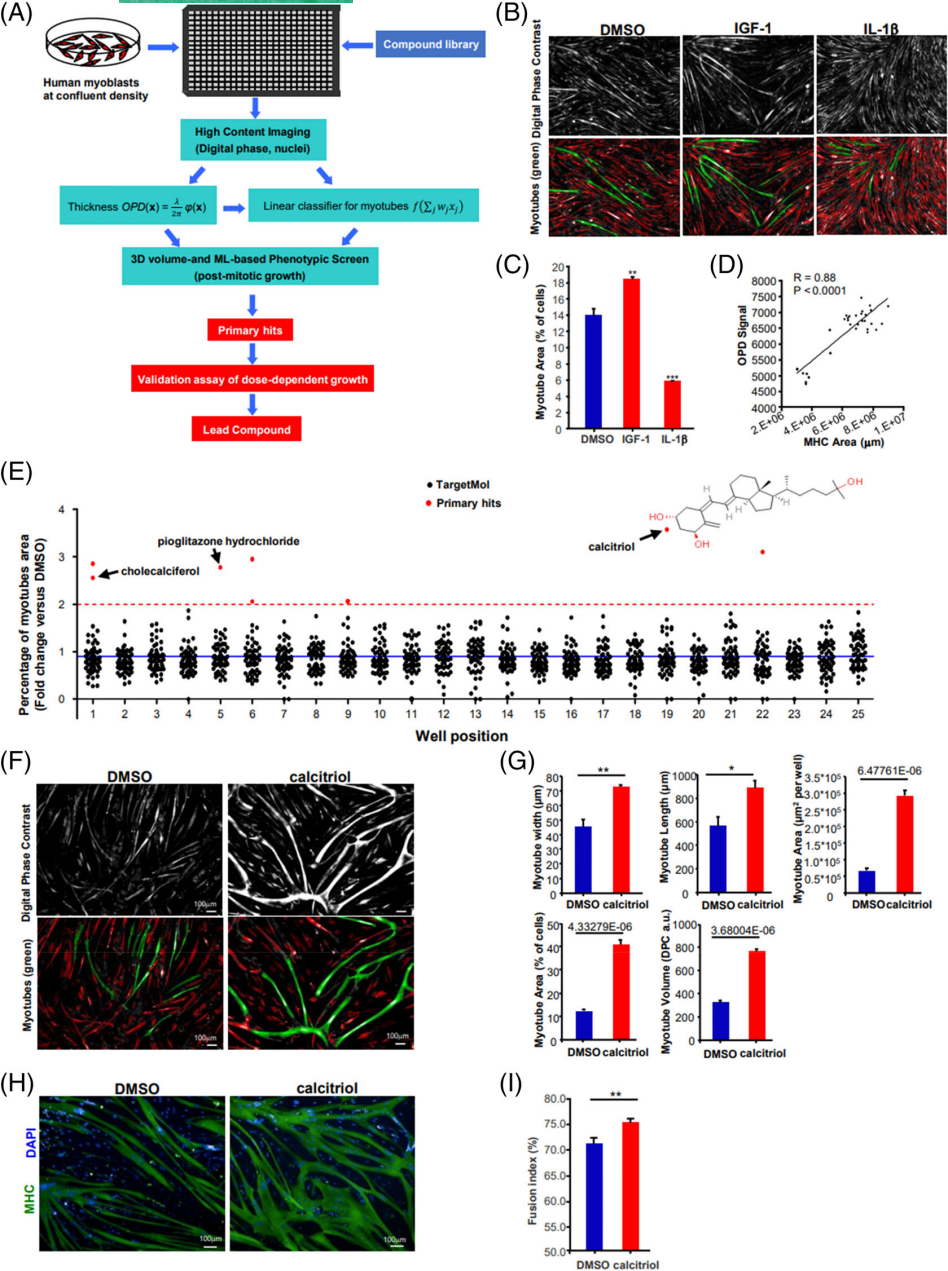
High‐throughput drug screens for postmitotic growth reveal calcitriol and cholecalciferol as top hits. (A) Flowchart of a high‐throughput drug screening process, with a primary screen based on ML‐driven recognition of human myoblast‐derived myotubes and their 3D volumes, followed by a validation assay of dose‐dependent effects. (B) Validation of the primary screen with the growth‐inducing IGF‐1 as a positive control, and the growth‐suppressive IL‐1β as a negative control. (C) Quantification of myotube area percentage after adding the growth‐inducing IGF‐1 as a positive control, and the growth‐suppressive IL‐1β as a negative control. (D) Correlation between the ML‐determined OPD signal and the MHC immunostaining area. *R*, Pearson's correlation coefficient. *p* < 0.0001. (E) Dot plot of high‐throughput screening data of the TargetMol library set used to identify new compounds that promote human myotube differentiation and maturation. The blue solid line delineates the mean response. The red dotted line indicates the positive hits, and eight primary positive hits were highlighted with red dots. Arrowhead: calcitriol. (F) Representative digital phase contrast (DPC) images for the estimated 3D volumes of DMSO‐ and calcitriol‐treated human myotubes at Day 7. (G) Myotube morphology (myotube width, length, area, percentage myotube area and volume) characteristics of human myotubes derived from myoblasts treated with calcitriol, relative to the DMSO vehicle control. *N* = 20 wells. (H) Myotube maturation as indicated by immunostaining for MHC (green) protein in human myotubes derived from myoblasts treated with calcitriol, relative to the DMSO vehicle control. Nuclei were counterstained with Hoechst (blue). (I) Fusion index for calcitriol‐ and DMSO‐treated myotubes. *N* = 20 wells. 3D, three‐dimensional; DMSO, dimethyl sulfoxide; MHC, myosin heavy chain; ML, machine learning; OPD, optical thickness distribution

Our goal was to establish a reliable and high‐throughput screening strategy for identifying drugs that boost human skeletal muscle postmitotic growth (Figure 1A). First, we treated primary human skeletal myoblasts that had exited mitosis with a large metabolite compound and small molecule library. Then, we analysed the cells using Digital Phase imaging and ML algorithms, to estimate 3D cell volumes in a high‐content, high‐throughput analysis system. Digital phase image construction uses a computational strategy to generate quantitative phase images with multiple red LED‐based brightfield images. The computational algorithm calculates the rate of change in light intensity distribution between different brightfield images at different focal planes, to obtain the refraction‐induced phase shift intensities of the specimen.

$$\mathrm{OPD}\left(x\right)=\frac{\lambda }{2\pi }\;\varphi \left(x\right),$$

that is, optical thickness is proportional to the phase shift intensity at point **x**.

$$\text{Volume}=n\oint \oint \mathrm{OPD}\left(x\right)\;\mathrm{dS},$$

assuming refractive index *n* is the same throughout all myotubes.

Pseudocolour rendering of the specimen phase shift intensity reveals speckle‐free and high‐contrast surface details on the myotubes and provides an accurate profile of the optical thickness distribution (OPD) function, which can be calculated directly from the quantified phase shifts. Contour integrals of the OPD over each myotube could then give accurate estimates of the relative 3D volume of each myotube.

For automated segmentation of each myotube's contour, we applied ML algorithms based on the Harmony PhenoLOGIC linear classifier module onto the speckle‐free, digital phase images. Threshold values for various morphological parameters were obtained by data training and optimization of the training cost function, based on the linear classifier equations:

$$y=f\left(w\times x\right)=f\left(\sum_{j}w_{j}x_{j}\right),$$

where *w* is a weight vector for *j* parameters that is learned from a set of labelled training samples, and *f* is a threshold function, which maps all values of w×x above a certain threshold to the myotube Class 1 and all other values to the nonmyotube Class 0, that is

$$f\left(x\right)=\left\{\begin{array}{c} 1\;\text{if}\;w\times x>\theta ,\\ 0\;\text{otherwise}. \end{array}\right.$$

After training the linear classifier, our ML algorithm extracted >200 multivariate features, with five features (including the OPD) weighted most heavily to efficiently separate multinucleated, hypertrophic myotubes from other types of myoblast‐derived myocytes (Figure 1A). To validate our ML algorithm, we compared the result given by ML with the analysis result by an experienced researcher, and the outcome was highly consistent. Moreover, our linear classifier's segmentation of multinucleated myotubes could robustly assess the progrowth phenotype of the anabolic growth‐inducing factor IGF‐1, and the anti‐growth phenotype of the inflammatory growth‐suppressive cytokine IL‐1β (Figures 1B,C). Furthermore, we observed a strong correlation between the ML‐determined OPD signal and the area of immunostained MHC+ myotubes (*R* = 0.88, *p* < 0.0001; Figure 1D). In addition, the ML analysis correctly identified postmitotic myotubes and excluded all mitotic Ki67+ muscle cells (Figure S1A). These findings indicate that our platform's 3D volume‐ and ML‐based quantification of myotubes could reliably assess postmitotic growth.

For the high‐throughput drug screen, we seeded primary human myoblasts onto 384‐well plates at high density, and screened natural small molecule compounds from the TargetMol library (Figure 1E). Primary hits for postmitotic growth were selected using the 3D volume‐ and ML‐based recognition of myotubes, and calculating the fold change in myotube percentage, relative to that of DMSO vehicle. The top primary hit was calcitriol (1,25(OH)2D3) or the bioactive form of vitamin D3 (Figure 1E). Pioglitazone, one of the other primary hits, has been shown to be a PPARg agonist that significantly improved skeletal muscle functions in a reserpine‐induced fibromyalgia rat model.^27^ Another primary hit, cholecalciferol, is an inactive precursor of calcitriol which requires sequential hydroxylation in the liver and then the kidney before it binds avidly to the vitamin D receptor,^28^ and has also been shown to improve muscle strength and performance in athletes and old adults.^29^, ^30^ The identification of pioglitazone and cholecalciferol as positive hits further supported the validity of our primary screening and data analysis, although the identification of both calcitriol and cholecalciferol despite their vastly different *K*_d_'s for VDR (3.8 pM and >20 mM, respectively) also suggested that VDR‐independent mechanisms might be operating.^28^

### 3.2 The dose‐dependent effect of calcitriol on human myotube maturation

Candidate hits were titrated to identify drugs that induced postmitotic growth in a dose‐dependent fashion and increased myogenic marker expression. To verify the effect of calcitriol, the top hit, on skeletal muscle growth, we set up titrations from 0.1 nM to 10 μM for a validation assay of dose‐dependent cell growth and muscle‐specific marker expression. Compared to the DMSO controls, calcitriol showed a clear dose‐dependent effect on the percentage of myotubes, and the maximum percentage was attained at a concentration of 0.1uM (Figure S1B). Importantly, the mean myotube 3D volume was significantly higher in the calcitriol group than the DMSO control group (Figure 1F,G). Other parameters such as myotube width, length, total area and myotube percentage area, were also significantly higher in the calcitriol group (Figure 1G). These observations showed that calcitriol dramatically promoted human myotube hypertrophy. Myotube terminal differentiation was further estimated by the fusion index, based on MHC staining at Day 7 (Figure 1H). Compared with the DMSO vehicle control group (70.80% fusion), calcitriol‐treated myoblasts differentiated into multinucleate myotubes to a slightly higher degree (75.08% fusion, Figure 1I). Similarly, mRNA profiling of myogenesis markers also showed that calcitriol induced the expression of myogenesis markers such as ACTA1 and MYH7 at Day 4 (Figure S1C). These results strongly suggest that calcitriol mainly promotes myotube growth and maturation, but also promotes myotube terminal differentiation. Based on the findings above, we chose calcitriol as the final lead compound.

### 3.3 Effects of calcitriol on myoblast proliferation, myotube growth and adhesion

**FIGURE 2:**
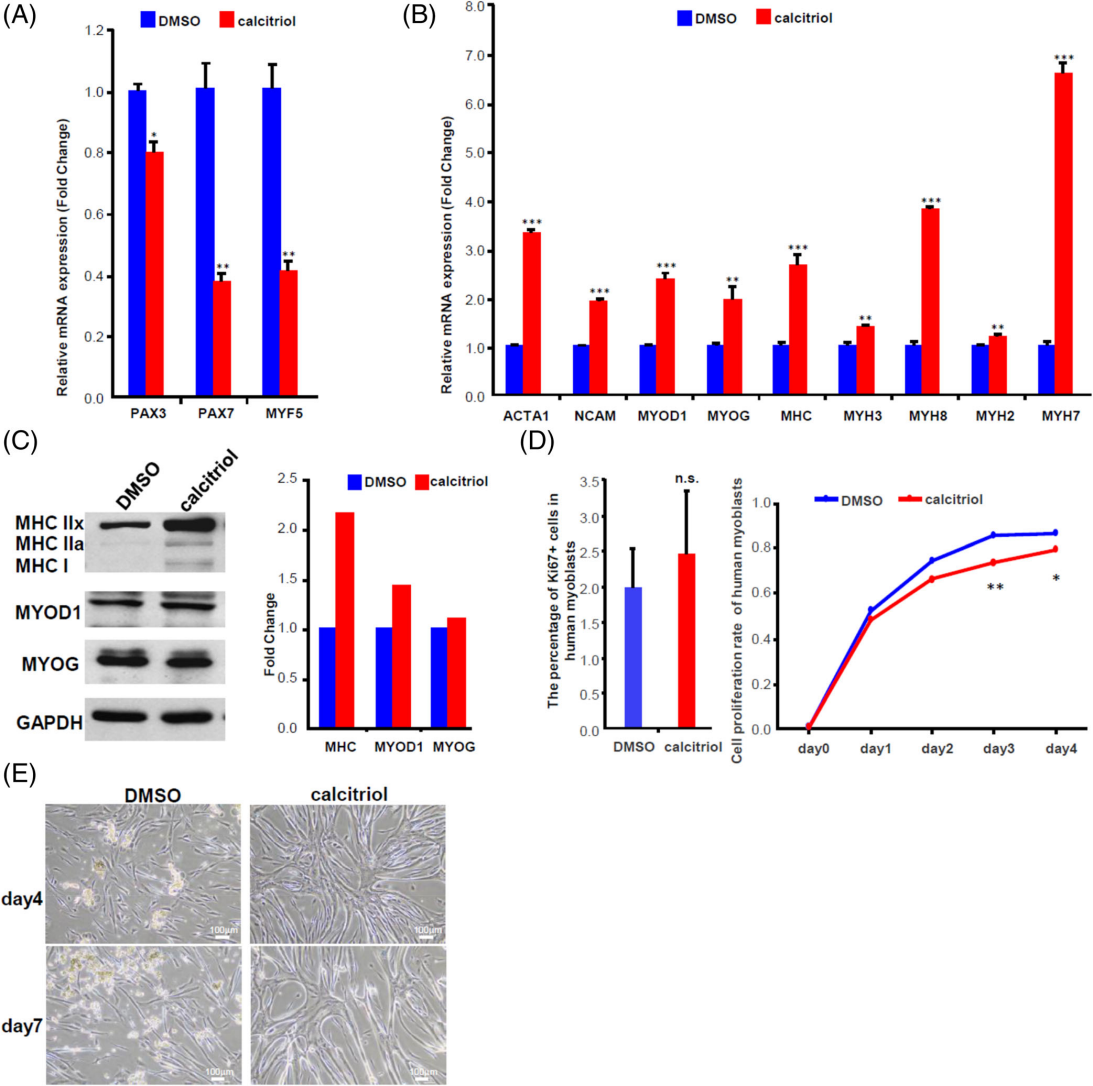
Calcitriol functionally promotes human muscle postmitotic growth and maturation. (A and B) Quantitative RT‐PCR for myogenic markers in DMSO‐ and calcitriol‐treated HSKM‐derived myotubes. (A) Undifferentiated myoblast marker genes' expression; (B) Myotube differentiation marker genes' expression. *p <0.05, **p <0.01, ***p <0.001, *n* = 3 individual experiments. (C) Western blot quantification of myosin heavy chain (MHC) subtypes, MYOD1, MYOG and GAPDH protein abundance in human myotubes treated with DMSO vehicle or 100 nM of calcitriol for 72 h. (D) Myoblast proliferative capacity was evaluated by staining and calculating the proportion of Ki67+ cells at Day 5, after treatment with DMSO vehicle or 100 nM calcitriol at Day 1. Myoblast‐myocyte proliferation was measured by counting the total DAPI+ cell numbers daily for 5 days, *n* = 3 individual experiments. (E) Representative images of cell delamination versus adhesion in human myotubes treated with DMSO vehicle or 100 nM calcitriol, at Days 4 and 7. DMSO, dimethyl sulfoxide; RT‐PCR, real‐time polymerase chain reaction

To confirm calcitriol's abilities to regulate proliferation, growth and differentiation, we performed mRNA profiling of calcitriol‐ and DMSO‐treated myotubes for a large variety of myoblast and myotube biomarkers. Calcitriol significantly downregulated the proliferative myoblast markers *PAX3*, *PAX7* and *MYF5* (Figure 2A), and significantly upregulated *MYOD1*, *MYOG* and other downstream biomarkers of postmitotic myotubes such as *ACTA1*, *NCAM1*, *MHC*, *MYH3*, *MYH8*, *MYH2* and *MYH7*, compared to the DMSO controls (Figure 2B). Furthermore, when we performed Western blot analysis for muscle‐specific protein biomarkers, we found that calcitriol dramatically increased the three MHC subtypes: MHC I, IIa and IIx (Figure 2C), when compared to DMSO group. These observations further support our observations that calcitriol has the ability to improve human myotube differentiation and maturation.

Most of the extant studies suggested that cholecalciferol inhibits mouse C2C12 and human myoblast proliferation.^31^, ^32^, ^33^ On the contrary, others have shown that calcitriol stimulates cell proliferation and inhibits myogenesis in undifferentiated chicken embryo myoblasts.^34^ To investigate the effect of calcitriol on human skeletal muscle myoblast proliferation, we performed cell counting and Ki67 immunofluorescence staining. Although the Ki67+ proportion was slightly increased in calcitriol‐treated myoblasts at Day 5, it was not statistically significant (Figure 2D). Moreover, cell counting revealed that calcitriol treatment suppressed primary human myoblast proliferation (Figure 2D). Thus, calcitriol promotes muscle cell growth without increasing cell proliferation.

It is also well‐known in the field that within 1–2 weeks of culture, most primary myotubes will undergo delamination^35^ and cell death due to imperfect metabolic conditions and inadequate extracellular matrix (ECM) biosynthesis in vitro. As expected, primary human myoblasts underwent delamination after just 5 days of differentiation, and flocculent aggregates of multinucleate myotubes were found floating in the culture medium by Day 7 (Figure 2E). In contrast, calcitriol‐treated myotubes showed significantly stronger ECM adhesion during differentiation (Figure 2E). Here, we further investigated if the adhesion effect of calcitriol would be abrogated by supplementing the culture surface with type I collagen during myotube differentiation. We found that calcitriol‐treated myotubes still showed stronger ECM on type I collagen‐coated culture wells at Day 5, whereas DMSO‐exposed myotubes were already undergoing delamination at Day 3 (Figure S2A). Moreover, both mRNA profiling and Western blots of MHC confirmed that calcitriol promotes myotube ECM adhesion, longer‐term growth and maturation even in the presence of type I collagen (Figures S2B and S2C).

### 3.4 Identification of protein targets for calcitriol using TPP

**FIGURE 3:**
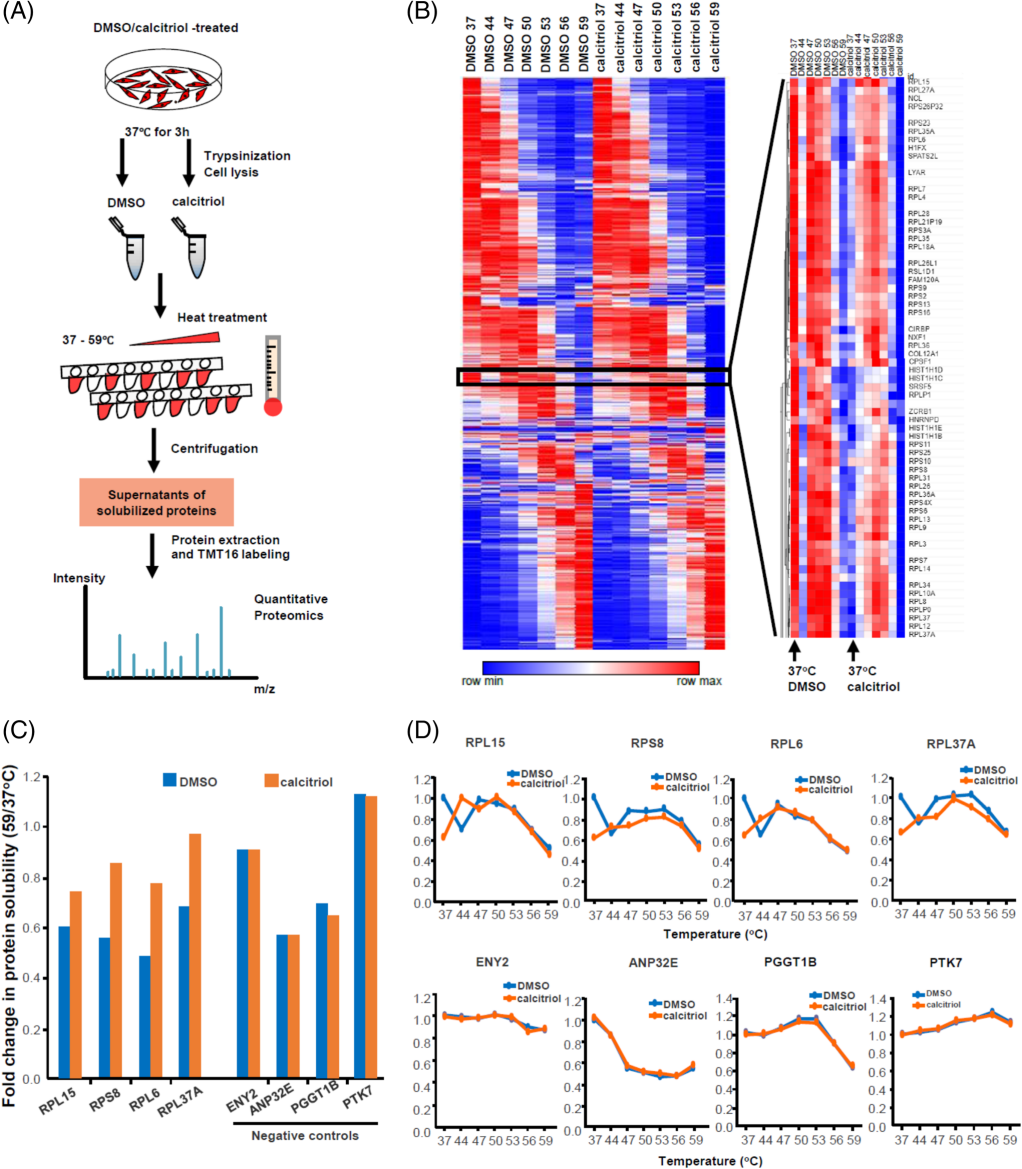
Unbiased identification of calcitriol's protein targets using thermal proteome profiling (TPP). (A) Schematic representation of the TPP‐temperature range experiments for unbiased discovery of drug‐binding targets. Myotubes were incubated with DMSO vehicle or 100 nM calcitriol for 3 h, before processing at different predetermined temperatures, labelling with TMT16 isotope labels for quantification, and proteomic analysis and quantification by LC–MS/MS. (B) Heatmap of protein solubility over the range of 37–59°C in human myotubes after exposure to DMSO vehicle or calcitriol for 3 h. Colour key indicates relative solubility, ranging from high (red) to low (blue). (C) Ribosomal proteins RPL15, RPS8, RPL6 and RPL37A showed the greatest changes in solubility at 59 versus 37°C after calcitriol treatment (orange), relative to DMSO vehicle treatment (blue). In comparison, ENY2, ANP32E, PGGT1B and PTK7 showed no changes in solubility. (D) Representative protein solubility/stability curves for the ribosomal proteins RPL15, RPS8, RPL6 and RPL37A over a temperature range of 37–59°C. In comparison, ENY2, ANP32E, PGGT1B and PTK7 showed no differences across the temperature range after calcitriol treatment. DMSO, dimethyl sulfoxide; LC–MS, liquid chromatography–mass spectrometry

The identification of both cholecalciferol and calcitriol in our primary screen, which have vastly different binding affinities *K*_d_'s for VDR,^28^ and the fact that VDR is only weakly expressed in adult muscle,^24^ suggested that VDR‐independent mechanisms might be operating. To identify novel binding targets of calcitriol in an unbiased manner, we used the latest MS‐based techniques for TPP. The thermal stability of drug‐binding versus nonbinding proteins was measured by quantification of the relative amounts of soluble proteins remaining in the supernatants at different temperatures (Figure 3A).

Hierarchical clustering of the TPP proteomic results showed that the thermal stability profiles were highly similar across the entire proteome with and without brief calcitriol treatment (Figure 3B), with the exception of 58 proteins in the middle of the heatmap, which displayed significantly different thermal stability profiles around 37–44°C after brief calcitriol treatment (Figure 3B). Intriguingly, most of these were ribosomal proteins, indicating that the ribosome proteins' solubility at 37–44°C were affected by brief treatment with calcitriol, and suggesting that the ribosome complex itself could bind to calcitriol (Figure 3B right). Representative ribosomal proteins' (RPL15, RPS8, RPL6 and RPL37A) solubility at 59 versus 37°C showed a significant difference after calcitriol treatment, compared to DMSO treatment (Figure 3C). In contrast, as negative comparative controls, the proteins ENY2, ANP32E, PGGT1B and PTK7 showed a variety of thermal stability trends, but little differences after calcitriol treatment (Figure 3C). When examined in greater detail, the solubility versus temperature (or “melting”) curves of the ribosomal proteins RPL15, RPS8, RPL6 and RPL37A were significantly different between calcitriol‐ and DMSO‐treated samples, whereas no differences in solubility were observed among the negative control proteins ENY2, ANP32E, PGGT1B and PTK7 over the whole TR (Figure 3D). These observations suggested that the ribosome complex binds directly to calcitriol to produce these thermal stability changes.

### 3.5 Calcitriol promotes protein translation through direct binding of the ribosome

**FIGURE 4:**
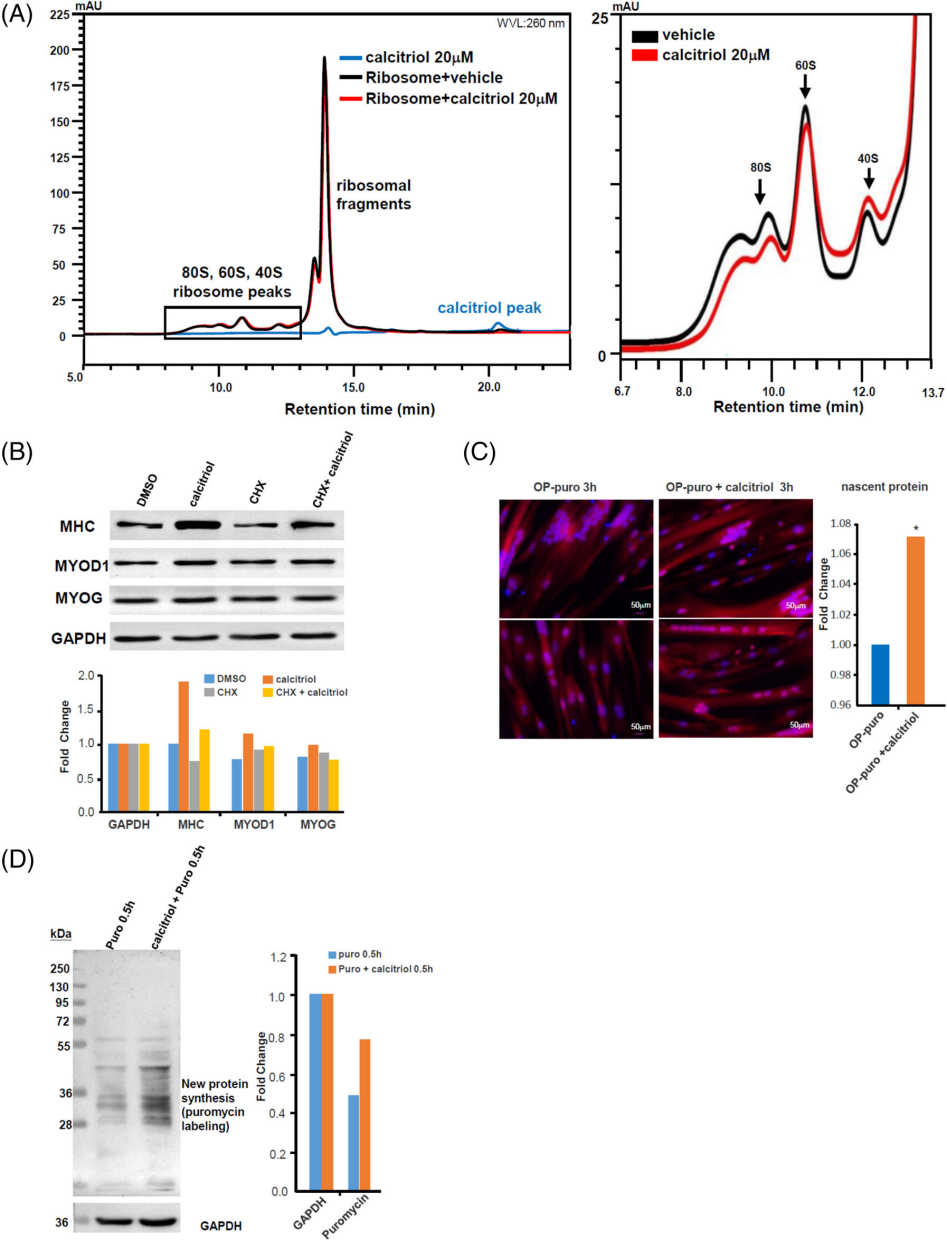
Calcitriol directly binds to ribosomes to promote protein translation in skeletal muscle cells. (A) Left. Representative Ribo Mega‐SEC chromatogram of the abundance of ribosomes (8–13 min) and ribosomal fragments (13–15 min) isolated and purified from human myotubes, treated with vehicle (black) or 20 μM calcitriol (red) in vitro, as analysed using uHPLC. Pure 20 μM calcitriol was ran as a comparison, with a peak at ~20.5 min (blue). Right. A zoom‐in on the Ribo Mega‐SEC chromatogram of the 80S ribosomes, 60S subunits and 40S subunits isolated and purified from human myotubes, treated with vehicle (black) or calcitriol (red) in vitro, as analysed using uHPLC. The horizontal axis represents retention time (min), and the vertical axis indicates the UV absorbance at 260 nm. (B) Representative Western blot quantification of MHC, MYOD1, MYOG and GAPDH protein abundance in human myotubes treated with the ribosomal inhibitor cycloheximide (CHX) for 16 h and treated with DMSO vehicle or calcitriol for 72 h. (C) Representative images of myotubes treated with O‐propargyl (OP)‐labelled puromycin to track nascent protein synthesis in myotubes after treatment with DMSO or calcitriol for 0.5 h. Nuclei were stained with Hoechst (blue). *N* = 3, *p* <0.05. (D) Representative Western blots for puromycin incorporation and GAPDH in human myotubes, in the absence or presence of calcitriol. DMSO, dimethyl sulfoxide

To confirm our TPP data analysis results with an orthogonal method, we performed a Ribo Mega‐SEC analysis of myotube ribosome proteins using uHPLC. Ribo Mega‐SEC is a recently developed approach for characterizing changes in ribosome assembly in response to drugs.^26^ Consistent with Yoshikawa et al.'s results, we observed unique peaks for 80S ribosome complexes, 60S subunits and 40S subunits at 8–13 min (Figure S3A). When calcitriol was added to the purified ribosomal protein components and incubated for 20 min at 4°C, the free calcitriol peak at ~20.5 min decreased significantly (Figure 4A left), and all uHPLC peaks for the 80S ribosomes, 60S subunits, 40S subunits showed distinct shifts (Figures S3B and 4A right). In particular, brief treatment of pure ribosomes with pure calcitriol reproducibly decreased the fast‐migrating 80S peak, and increased the slow‐migrating 40S peak (Figure 4A right). Unbound small molecules would have eluted separately from the ribosome peaks, as free calcitriol did at ~20.5 min, and should not affect the ribosome peaks at 8–13 min. Thus, these Ribo Mega‐SEC findings strongly support our conclusion that calcitriol directly binds to purified ribosome complexes isolated from human myotubes, and possibly keeps them in a loosened, activated state.

To further confirm if calcitriol increases human muscle protein translation activity through direct binding to ribosomes, we conducted Western blot analysis for MHC, MYOD1 and MYOG in myotubes, with and without the well‐established ribosomal translation inhibitor cycloheximide (CHX). Calcitriol significantly increased MHC protein expression, relative to the DMSO vehicle (Figure 4B). However, this increase was specifically abrogated by low concentrations of CHX that had little effect on MHC in the DMSO control, or other proteins regardless of DMSO or calcitriol treatment, indicating that calcitriol was upregulating MHC protein abundance by promoting ribosomal translation (Figure 4B). Cell morphology observations on myotube differentiation corroborated these findings (Figure S4). In addition, we also measured nascent protein synthesis by using a nonradioactive probe for protein incorporation of fluorescently‐labelled *O*‐propargyl ‐puromycin, in the absence or presence of calcitriol. Compared to the DMSO vehicle control, calcitriol stimulation significantly increased the incorporation of fluorescent puromycin after just 3 h of incubation, indicating that calcitriol can rapidly promote nascent protein synthesis in human myotubes (Figure 4C). Finally, Western blot analysis for puromycin incorporation further confirmed that calcitriol stimulation significantly increased nascent protein synthesis within 0.5 h (Figure 4D). These data further support our findings that calcitriol can rapidly promote protein translation in human skeletal muscle, well before the genomic effects of VDR‐dependent transcription can begin to take effect.

## 4 DISCUSSION

To restore tissue growth without increasing the risk for cancer during ageing, there is a need to identify small molecule drugs that can increase postmitotic growth without increasing cell proliferation. In our high‐throughput phenotypic drug screen for postmitotic anabolic growth, by using ML algorithms to estimate 3D cell volumes, vitamin D3 metabolites stood out as top hits. Vitamin D3 is known to modulate the physiology and function of multiple human tissues, including the skeletal muscles. Numerous epidemiological studies have revealed that vitamin D3 supplementation is able to improve muscle strength and physical performance in sarcopenia or elderly patients,^36^, ^37^, ^38^ but the mechanisms have remained unclear, especially since VDR expression is low in adult skeletal muscles. Some studies have suggested that vitamin D3 enhances muscle strength by increasing the type II myofibre area.^39^, ^40^ In our current study, we found that both calcitriol and cholecalciferol can increase postmitotic myotube growth, and that calcitriol can significantly increase both type I and IIa/IIx MHC protein levels, in part by directly increasing ribosomal protein synthesis. We speculate that such MHC proteins are disproportionately translated at high efficiency in muscles, because myosin is the most abundant protein in skeletal muscle.^41^

In our current study, calcitriol improved myotube differentiation and maturation but inhibited myoblast proliferation. Previous studies have shown that calcitriol can promote mouse C2C12 myoblast differentiation,^42^ as well as primary human myogenic differentiation.^33^ In contrast, calcitriol stimulates cell proliferation in undifferentiated chicken embryo myoblasts.^34^ This is not surprising, given that ribosomal translation is needed for both cell proliferation and differentiation. Our screen only purports to identify small molecules that can promote postmitotic growth in human myotubes, but does not exclude small molecules that can both promote myotube growth and myoblast proliferation in other species. Muscle development is known to vary from species to species, with direct and indirect effects on their energy metabolism, signalling pathways, transcription factors and miRNAs.^43^, ^44^

Calcitriol can stimulate protein synthesis in skeletal muscle through both VDR‐dependent and ‐independent mechanisms.^35^ Ryan et al. showed that calcitriol can modulate VDR‐dependent transcription of mRNAs encoding ECM‐receptor proteins, muscle contraction, focal adhesion, and integrin signalling pathways.^19^ In our present study, calcitriol prevented myotube delamination from type I collagen‐coated surfaces, possibly by increasing ECM protein biosynthesis as well. Attempts to develop in vitro human muscle fibre models have long been hampered by premature human myotube delamination, and difficulties in sustaining long‐term human myotube culture in vitro hitherto.^45^ This has been a nontrivial problem in the field of muscle cell culture, and calcitriol's preventive effects on myotube delamination might represent a new strategy to address this problem for in vitro drug screening and tissue engineering.

Given that both cholecalciferol and calcitriol emerged in our high‐throughput screen, despite their disparate binding affinities for VDR, we were motivated to search for VDR‐independent targets of calcitriol, using TPP proteomics.^46^ Perrin et al. have previously used TPP to identify the targets of panobinostat in rat liver, lung, kidney and spleen, and revealed that ZNF512 binds to panobinostat.^46^ TPP also enabled the high‐throughput discovery of drug targets and downstream effectors,^47^ including transmembrane protein targets.^48^ In TPP, the temperature gradient gradually denatures and precipitates proteins irreversibly in general. But individual proteins with different structures, especially after binding small molecules, might have different thermal solubility or ‘melting’ curves.^13^, ^49^ While traditional approaches assumed most protein solubility or ‘melting’ curves follow a sigmoidal shape, deviations from sigmoidal curves have also been found to be very common,^50^, ^51^, ^52^ especially for protein complexes. Using hierarchical clustering and maximum fold change analyses, we identified many novel targets of calcitriol, of which an overwhelming majority are ribosomal proteins. Importantly, Ribo Mega‐SEC, puromycin incorporation and functional assays orthogonally confirmed the ribosome complex as a novel target of calcitriol, and verified that TPP data analysis is a reliable strategy for high‐throughput target discovery after ML‐based phenotypic screening.

Many previous studies have focused on ribosomal inhibitors such as the natural compounds puromycin and cycloheximide, which bind very tightly to lock ribosomes in certain conformations, but none have uncovered ribosomal agonists hitherto. To our knowledge these are the first archetypal members of small molecule ribosome agonists, which could find applications in the treatment of muscle degenerative diseases of ageing. In comparison, proteasome modulators, which also regulate steady state levels of protein abundance, have seen some success with muscle atrophy due to sepsis or denervation, but largely failed to reverse sarcopenia and cachexia in vivo.^53^ Indeed, previous studies have already shown that vitamin D3, through genomic or nongenomic effects, can also enhance the development and growth of brain, bone, immune, ovarian follicle, skin, hair and sperm cells.^54^, ^55^, ^56^, ^57^, ^58^, ^59^, ^60^ Our results suggest that ribosome agonists might provide an alternative approach for preventing and treating tissue degeneration in the future.

## CONFLICTS OF INTEREST

The authors declare no conflicts of interest.

## AUTHOR CONTRIBUTIONS

Zongmin Jiang and Ng Shyh‐Chang designed the study. Zongmin Jiang, Liping Zhang, Ziyue Yao, Wenhua Cao, Shilin Ma, Yu Chen and Lu Guang performed the experiments. Zongmin Jiang, Liping Zhang, Ziyue Yao, Wenhua Cao, Zipeng Zheng, Chunwei Li, Kang Yu and Ng Shyh‐Chang analysed the data. Zongmin Jiang and Ng Shyh‐Chang wrote the paper.

## Supporting information


**FIGURE S1 Dose‐dependent effects of calcitriol on human myotube maturation.** (A) Representative OPD and myosin heavy chain (MHC, green) protein immunostaining images in human myotubes derived from myoblasts treated with calcitriol, relative to the DMSO vehicle control. The proliferative mitotic muscle cells were stained by Ki67 (Red). Nuclei were counterstained with Hoechst (blue). (B) Representative digital phase contrast (DPC) images for the estimated %myotube area of calcitriol‐ and DMSO‐treated human myotubes at day 7. (C) Quantitative RT‐PCR for myogenic markers' gene expression in calcitriol‐ and DMSO‐treated human myotubes. *p <0.05, **p <0.01, ***p <0.001, n = 3 individual experiments.
**FIGURE S2. Calcitriol prevents myotube delamination and promotes myotube maturation on collagen type I ECM.** (A) Representative images of human myotube growth after treatment with DMSO vehicle or calcitriol on collagen type I‐coated culture surface for 5 days. (B) Quantitative RT‐PCR for myogenic markers in human myotubes treated with DMSO or calcitriol on collagen type I‐coated culture surface. *p <0.05, **p <0.01, ***p <0.001, n = 3 individual experiments. (C) Western blot quantification of MHC, MYOD1, MYOG and GAPDH protein abundance in human myotubes exposed to DMSO vehicle or calcitriol on collagen type I‐coated culture surface.
**FIGURE S3. Calcitriol directly binds to ribosomes.** (A, B) Representative Ribo Mega‐SEC chromatogram of the abundance of purified polysomes, 80S ribosomes, 60S subunits and 40S subunits isolated from human myotubes, treated with DMSO vehicle (A) or calcitriol (B) in vitro, as analysed using uHPLC. X‐axis represents retention time, and Y‐axis indicates the UV absorbance at 260 nm. Table describes the peak area and UV signal values corresponding to each peak.
**FIGURE S4. Calcitriol effects on post‐mitotic growth depends on ribosome activation.** Representative images of myotube growth after treatment with CHX for 16 h and then treatment with DMSO vehicle or calcitriol for 72 h.

## Data Availability

The authors declare that all the data supporting the findings of this study are available within the article and from the corresponding authors upon reasonable request.
