# Supplementary material for: Machine learning‐based phenotypic screening for postmitotic growth inducers uncover vitamin D3 metabolites as small molecule ribosome agonists
Source: Cell Prolif. 2022 Apr 12;55(5):e13214. doi: 10.1111/cpr.13214 (PMC9136510; doi:10.1111/cpr.13214)
Supplement: Supplementary file 1 — FIGURE S1 Dose‐dependent effects of calcitriol on human myotube maturation. (A) Representative OPD and myosin heavy chain (MHC, green) protein immunostaining images in human myotubes derived from myoblasts treated with calcitriol, relative to the DMSO vehicle control. The proliferative mitotic muscle cells were stained by Ki67 (Red). Nuclei were counterstained with Hoechst (blue). (B) Representative digital phase contrast (DPC) images for the estimated %myotube area of calcitriol‐ and DMSO‐treated human myotubes at day 7. (C) Quantitative RT‐PCR for myogenic markers' gene expression in calcitriol‐ and DMSO‐treated human myotubes. *p <0.05, **p <0.01, ***p <0.001, n = 3 individual experiments. FIGURE S2. Calcitriol prevents myotube delamination and promotes myotube maturation on collagen type I ECM. (A) Representative images of human myotube growth after treatment with DMSO vehicle or calcitriol on collagen type I‐coated culture surface for 5 days. (B) Quantitative RT‐PCR for myogenic markers in human myotubes treated with DMSO or calcitriol on collagen type I‐coated culture surface. *p <0.05, **p <0.01, ***p <0.001, n = 3 individual experiments. (C) Western blot quantification of MHC, MYOD1, MYOG and GAPDH protein abundance in human myotubes exposed to DMSO vehicle or calcitriol on collagen type I‐coated culture surface. FIGURE S3. Calcitriol directly binds to ribosomes. (A, B) Representative Ribo Mega‐SEC chromatogram of the abundance of purified polysomes, 80S ribosomes, 60S subunits and 40S subunits isolated from human myotubes, treated with DMSO vehicle (A) or calcitriol (B) in vitro, as analysed using uHPLC. X‐axis represents retention time, and Y‐axis indicates the UV absorbance at 260 nm. Table describes the peak area and UV signal values corresponding to each peak. FIGURE S4. Calcitriol effects on post‐mitotic growth depends on ribosome activation. Representative images of myotube growth after treatment with CHX for 16 h and then treatment with DMSO vehicle [file CPR-55-e13214-s001.docx]

**Supplemental figures**


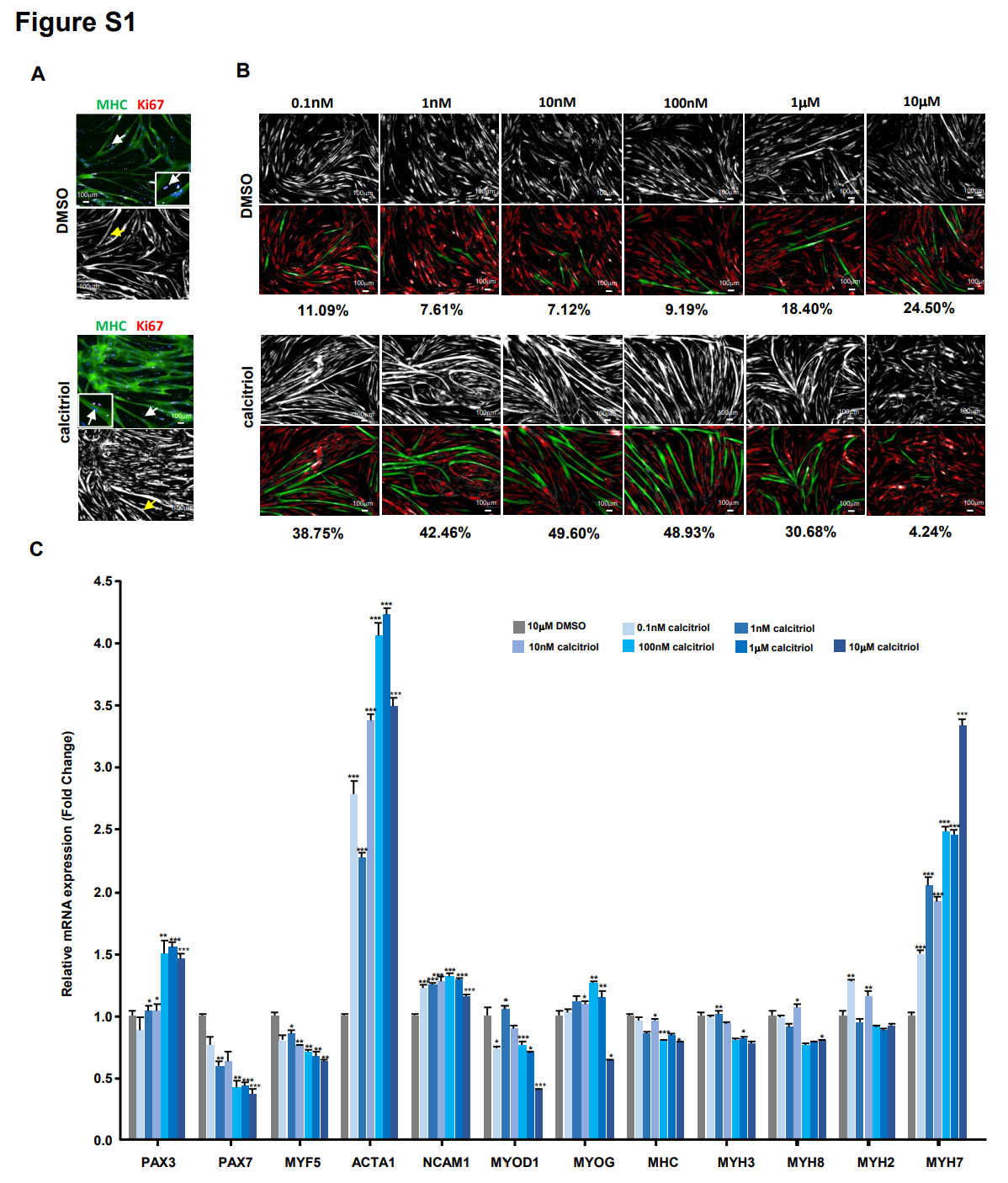


**Supplemental Figure 1. Dose-dependent effects of calcitriol on human myotube maturation.** (A) Representative OPD and myosin heavy chain (MHC, green) protein immunostaining images in human myotubes derived from myoblasts treated with calcitriol, relative to the DMSO vehicle control. The proliferation/mitosis muscle cells were stained by Ki67 (Red). Nuclei were counterstained with Hoechst (blue). (B) Representative digital phase contrast (DPC) images for the estimated %myotube area of calcitriol- and DMSO-treated human myotubes at day 7. (C) Quantitative RT-PCR for myogenic markers’ gene expression in calcitriol- and DMSO-treated human myotubes. *p < 0.05, **p < 0.01, ***p < 0.001, n = 3 individual experiments.


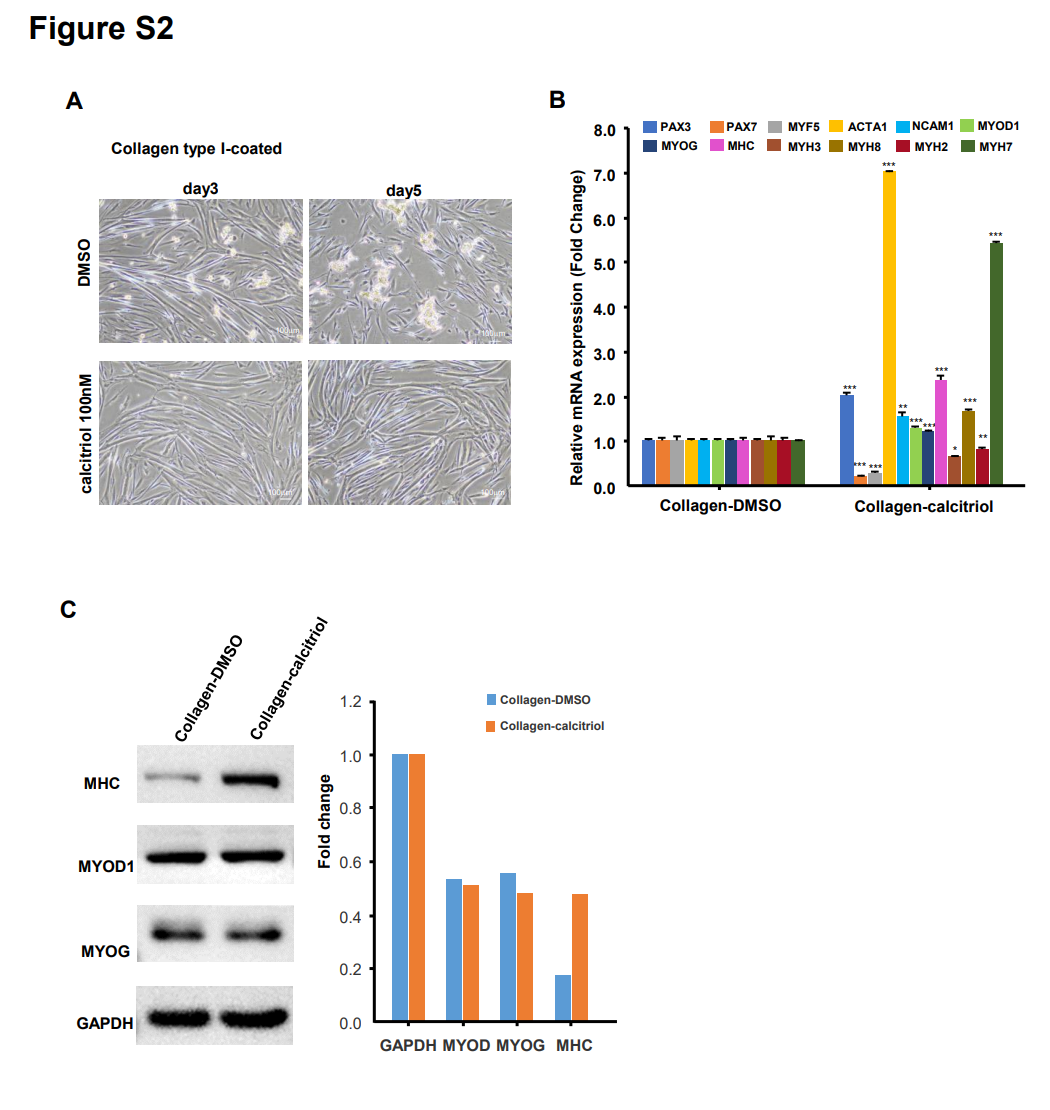


**Supplemental Figure 2. Calcitriol prevents myotube delamination and promotes myotube maturation on collagen type I ECM.** (A) Representative images of human myotube growth after treatment with DMSO vehicle or calcitriol on collagen type I-coated culture surface for 5 days. (B) Quantitative RT-PCR for myogenic markers in human myotubes treated with DMSO or calcitriol on collagen type I-coated culture surface. *p < 0.05, **p < 0.01, ***p < 0.001, n = 3 individual experiments. (C) Western blot quantification of MHC, MYOD1, MYOG and GAPDH protein abundance in human myotubes exposed to DMSO vehicle or calcitriol on collagen type I-coated culture surface.


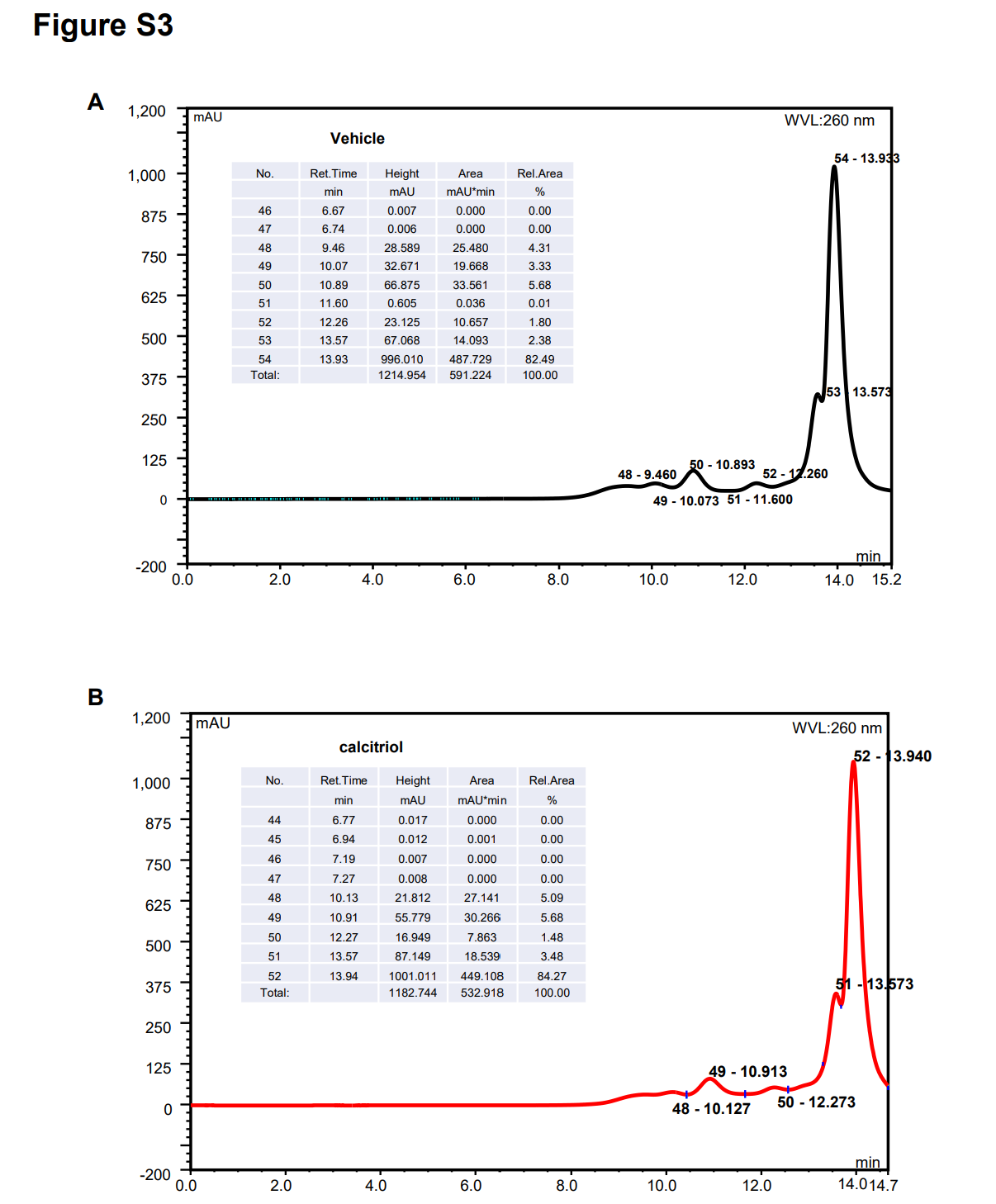


**Supplemental Figure 3. Calcitriol directly binds to ribosomes.** (A, B) Representative Ribo Mega-SEC chromatogram of the abundance of purified polysomes, 80S ribosomes, 60S subunits and 40S subunits isolated from human myotubes, treated with DMSO vehicle (A) or calcitriol (B) in vitro, as analyzed using uHPLC. X-axis represents retention time, and Y-axis indicates the UV absorbance at 260nm. Table describes the peak area and UV signal values corresponding to each peak.


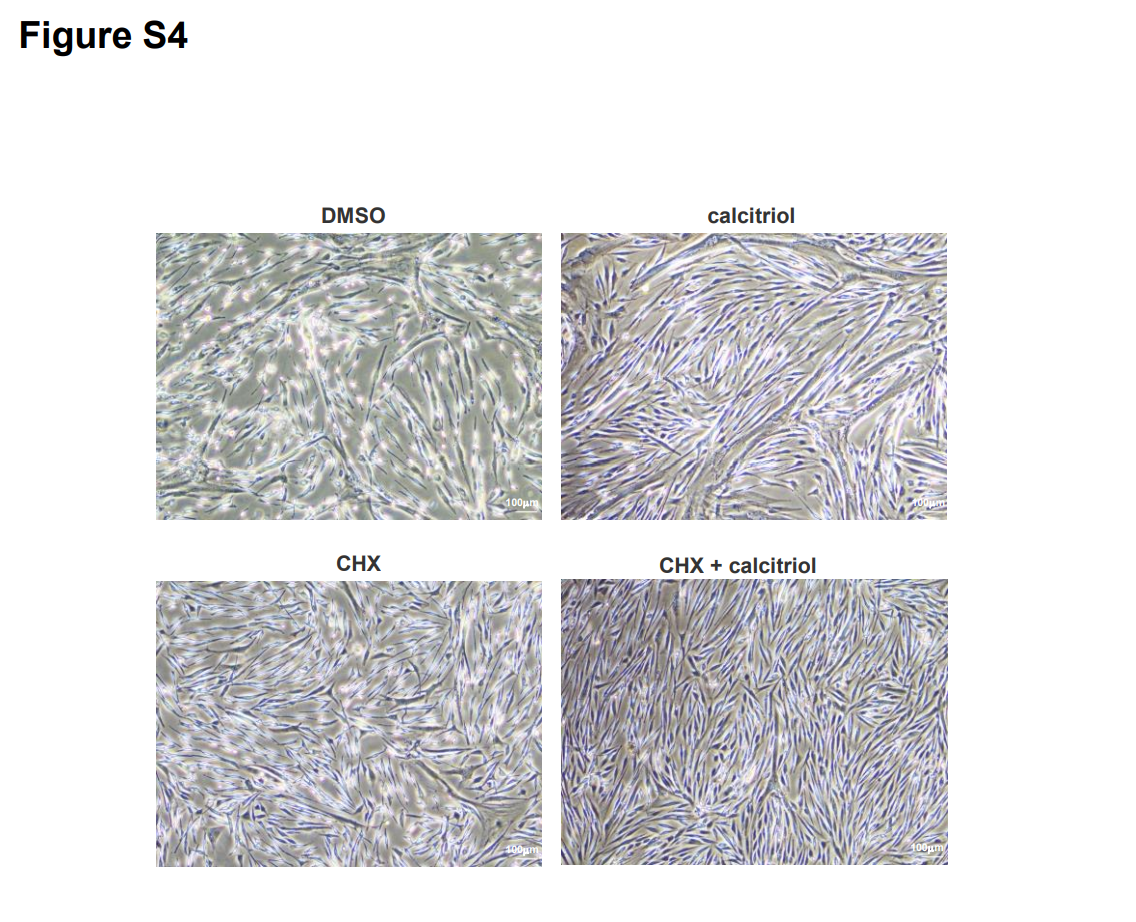


**Supplemental Figure 4. Calcitriol effects on post-mitotic growth depends on ribosome activation.** Representative images of myotube growth after treatment with CHX for 16h and then treatment with DMSO vehicle or calcitriol for 72h.
